# The journey continues in *Clinical Diabetes and Endocrinology*

**DOI:** 10.1186/s40842-018-0067-2

**Published:** 2018-07-31

**Authors:** Ariel Barkan, Roma Gianchandani, Meng H. Tan

**Affiliations:** Division of Metabolism, Endocrinology & Diabetes, Department of Internal Medicine, University of Michigan, Ann Arbor, MI 48106 USA

In 2015, BMC launched *Clinical Diabetes and Endocrinology* (CDE), a peer reviewed, open access academic journal dedicated to improving the care of people affected by diabetes and endocrine disorders. The University of Michigan’s Division of Metabolism, Endocrinology & Diabetes has been a partner in this journal since its inception, and has a longstanding commitment to advancing global interest in diabetes and endocrinology, as both Maturity Onset Diabetes of the Young (MODY) by Dr. Stefan Fajans [1] and Primary Aldosteronism by Dr. Jerome Conn [2] were both first described at Michigan. National and international collaborations including Michigan and multiple other outstanding academic centers have led to important contributions to the fields of GI hormones, Cushing’s syndrome, CNS control of the gonadal axis, obesity and insulin resistance, endocrine oncology, diabetes clinical practice, and more.

*CDE* was launched under the leadership of Dr. Meng H Tan as the inaugural Editor-in-Chief along with Editorial Board members from 19 nations in 5 continents. Thus far, the journal has published articles that have covered many important aspects of diabetes mellitus, thyroid, pituitary, and adrenal disorders, bone health, dyslipidemias, podiatry, diabetes education, peer-support, depression and lipodystrophy research, from case reports to clinical trials. Some of the most highly accessed articles in *CDE* provide clinicians with an overview of the literature on specific topics and improve their ability to handle these situations; these include

- Management of diabetes mellitus in patients with chronic kidney disease [3]
- Managing diabetes in the digital age [4]
- Thyroid nodule update on diagnosis and management [5]
- Hypophysitis: Evaluation and Management [6]

In July 2017, *CDE* reached its first major milestone, becoming indexed in PubMed; we’ve since seen the number of accesses to its published manuscripts steadily increase.

Given Dr. Tan’s impending retirement in July 2018, we are delighted to announce the passing of the torch to two new co-Editors-in-Chief: Drs. Ariel Barkan and Roma Gianchandani. Both are well-published clinician scientists who have served on the *CDE* Editorial Board from the beginning. Additionally, Dr. Tan will continue on at *CDE* as Editor Emeritus.

Both Drs. Barkan and Gianchandani are excited and enthusiastic about the future of *CDE*:

‘*This is a unique and exciting time in diabetes and endocrinology with unprecedented number of innovations in medications, technology and big data. We feel fortunate to be co-Editors-in-Chief of an open access platform with the ability to share these discoveries with colleagues around the world and improve patient care. We welcome comments and suggestions about the direction of this journal.”*

Going forward, *CDE* intends to open its pages, inviting colleagues from around the world to share their new knowledge and findings related to diabetes and endocrine diseases via our platform. We encourage submissions from colleagues at national and international diabetes/endocrinology centers as well as other specialties that have innovative studies related to diabetes and endocrinology. Cross-pollination and collaboration of ideas is the direction of medicine today. Together with our global Editorial Board members and a streamlined and well-organized review process, *CDE* intends to grow and make a significant contribution to the world of diabetes and endocrinology.
